# Treadmill prehabilitation attenuates jejunal inflammation and enhances cytoprotective protein expression in murine gut ischemia–reperfusion

**DOI:** 10.1371/journal.pone.0362208

**Published:** 2026-11-04

**Authors:** Kazuya Higashizono, Kazuya Takahashi, Kazuhiko Fukatsu, Tomonori Narita, Haruka Takayama, Midori Noguchi, Satoshi Murakoshi, Yasuyuki Seto

**Affiliations:** 1 Department of Gastrointestinal Surgery, Graduate School of Medicine, The University of Tokyo, Tokyo, Japan; 2 Department of Surgical Metabolism and Operating Room Management, Graduate School of Medicine, The University of Tokyo, Tokyo, Japan; 3 School of Nutrition and Dietetics, Faculty of Health and Social Work, Kanagawa University of Human Services, Yokosuka, Kanagawa, Japan; University of Minnesota Medical School, UNITED STATES OF AMERICA

## Abstract

**Introduction:**

Whether preoperative exercise protects the intestine itself is unclear. We hypothesized that treadmill prehabilitation acts on the intestine as ischemic preconditioning, and examined intestinal injury and cytokine responses after gut ischemia–reperfusion (I/R) and jejunal cytoprotective protein expression before ischemia.

**Methods:**

Two experiments were conducted in male C57BL/6J mice. In Experiment 1, mice were randomly allocated to a sedentary or an exercise group; exercised mice completed 1 week of acclimation and 3 weeks of treadmill running. One day after the final session, the superior mesenteric artery was occluded for 45 min. Jejunal and ileal cytokines were measured in 24 mice, and mucosal injury was graded by the Chiu classification in 22 mice, at 3 or 6 h after reperfusion. In Experiment 2, separate cohorts completed 0 (SED, n = 5), 1 (EX1, n = 5), 2 (EX2, n = 4) or 3 (EX3, n = 5) weeks of running, and jejunal HSP70, Bcl-2 and p62 were quantified by western blotting without I/R.

**Results:**

Injury scores were lower in exercised mice (jejunum at 3 h, 1.10 ± 0.22 vs 2.60 ± 0.50; main effect of exercise, P = 0.0025 [jejunum] and P < 0.0001 [ileum]). Jejunal IL-12 at 3 h (adjusted P = 0.042) and IL-6 at 6 h (adjusted P = 0.015) were lower in exercised mice, whereas in the ileum a main effect of exercise was found for IL-12 (P = 0.037) without adjusted between-group differences. HSP70/β-actin was higher in EX1–EX3, and p62/β-actin lower in EX2–EX3, than in SED (Bonferroni-corrected threshold, P < 0.017). Training duration was associated positively with HSP70/β-actin (R² = 0.55, P = 0.0003) and Bcl-2/β-actin (R² = 0.31, P = 0.014) and negatively with p62/β-actin (R² = 0.31, P = 0.014); group comparisons for Bcl-2 were not significant.

**Conclusion:**

Treadmill prehabilitation was accompanied by less mucosal injury and a smaller early jejunal inflammatory response after gut I/R, and the jejunum before ischemia differed in cytoprotective and autophagy-related protein expression according to training duration, consistent with ischemic preconditioning of the intestine.

## Introduction

Preoperative exercise, or prehabilitation, may reduce postoperative complications by improving physical reserve [1–5]. We have previously reported that treadmill running improves survival and attenuates organ injury, systemic oxidative stress, and inflammatory responses after gut ischemia–reperfusion (I/R) in mice [6,7]. Gut I/R occurs during shock, aortic surgery, trauma, and major surgery with severe hemorrhage, and carries substantial morbidity and mortality [7,8]. How exercise protects the intestine itself, however, is not known.

Repeated brief ischemia renders an organ tolerant to a subsequent sustained ischemic insult in the heart [9] and the kidney [10], and this tolerance has been associated with induction of heat shock proteins and autophagy-related responses [10–12]. In humans, intestinal blood flow falls during exercise and recovers afterwards [13,14]. Repeated exercise may therefore subject the intestine to brief, repeated ischemia and reperfusion, and the oxidative stress generated by ischemia and reperfusion is itself a known inducer of autophagy [15]. We therefore hypothesized that treadmill prehabilitation acts on the intestine as ischemic preconditioning, conferring ischemic tolerance through the induction of cytoprotective proteins.

This hypothesis predicts that the expression of cytoprotective proteins in the trained intestine is already increased before injury, in proportion to the number of training sessions. Experiment 1 confirmed in the present cohorts that gut I/R produced mucosal injury and that this injury was attenuated by exercise, and compared local cytokine concentrations in the jejunum and ileum. Because the exercise-associated cytokine difference was more evident in the jejunum, Experiment 2 tested this prediction by measuring jejunal HSP70, Bcl-2, and p62 expression in intestine that had not undergone I/R, using training durations of 1, 2, and 3 weeks (three, six, and nine sessions).

## Materials and methods

### Animals

Five-week-old male C57BL/6J mice weighing 18–21 g were purchased from Charles River Laboratories, Inc. (Yokohama, Japan). Only male mice were used; therefore, possible sex-related differences were not assessed. Mice were housed under a 12:12-h light/dark cycle (lights on at 8:00 AM) in a temperature-controlled environment and were provided standard chow (AIN-93G; Oriental Yeast Co., Ltd., Tokyo, Japan) and water ad libitum. The study protocol was reviewed and approved by the Animal Care Committee of The University of Tokyo (approval no. P19-047). Mice were allocated using a computer-generated randomization sequence. Investigators were blinded to group allocation during cytokine assays, histological grading, and densitometric quantification. No animals were excluded from any analysis. Mice were euthanized under deep anesthesia by exsanguination via cardiac puncture in accordance with institutional guidelines. Humane endpoints were predefined, and animals were monitored at least daily according to the approved protocol.

### Experiment 1

Mice were randomly allocated to a sedentary or an exercise group. The treadmill regimen was based on our previous study [7]. Exercised mice completed 1 week of acclimation (12 m/min, 0° incline, 15 min every other day, three sessions/week), followed by 3 weeks of treadmill running (12 m/min, 0° incline, 60 min/session, three sessions/week). Sedentary mice remained in their cages without treadmill running. One day after the final session, all mice underwent gut I/R and were euthanized at 3 or 6 h after reperfusion.

Not every animal was used for every measurement. Jejunal and ileal cytokine concentrations were measured in 24 mice (sedentary, n = 6 and exercise, n = 6 at each time point). Histological injury was graded in 22 mice (sedentary, n = 6 and exercise, n = 6 at 3 h; sedentary, n = 5 and exercise, n = 5 at 6 h). Fifteen animals contributed to both measurements. The animals used for each measurement were selected at random from this experiment.

### Gut ischemia–reperfusion

The mice were anesthetized with a subcutaneous injection of a mixture of ketamine (100 mg/kg) and xylazine (10 mg/kg). Gut I/R was performed according to a previous study [7]. Briefly, after a 1 cm midline laparotomy, the superior mesenteric artery (SMA) was identified. The SMA was clamped with a non-crushing microvascular clip (YASARGIL Aneurysm Clip System; AESCULAP®-a B. Braun brand, cat. no. FT220T) for 45 min. The laparotomy incision was temporarily closed during occlusion, and the clip was removed after 45 min. Immediately after reperfusion, 1 mL of normal saline was injected subcutaneously for resuscitation, and the laparotomy was closed with a running 6−0 nylon suture. Mice were euthanized 3 or 6 h after reperfusion.

### Cytokine levels in the jejunum and ileum

Jejunal and ileal tissues were collected at each time point after exsanguination and divided into 10-mm segments. Samples combining oral and anal segments from each intestinal region were thawed and homogenized for 30 s at 4°C in 10 volumes of lysis buffer (0.75% NH4Cl, 10 mM Tris, and 5 mM EDTA) containing a protease inhibitor cocktail (Roche, cat. no. 11697498001). Homogenates were centrifuged at 14,000 rpm for 45 min at 4°C. Supernatant concentrations of IL-12, TNF-α, IFN-γ, MCP-1, IL-6, and IL-10 were quantified using the BD Cytometric Bead Array Mouse Inflammation Kit (BD Biosciences, cat. no. 552364), a multiplex bead-based immunoassay analyzed by flow cytometry. Values below the lower limit of detection were recorded as 0 pg/mL and retained as 0 in the analyses. Individual values are provided in S2 Data.

### Histological assessment of intestinal injury

Ten-millimeter segments from the middle portions of the jejunum and ileum were fixed in 10% formalin, embedded in paraffin, sectioned at a thickness of 4 μm, and stained with hematoxylin and eosin. Specimens for grading were selected at random from the fixed tissues of this experiment.

Mucosal injury was assessed using the injury scoring system of the Chiu classification [16], which is based on changes in the villi and glands of the intestinal mucosa: grade 0, normal mucosal villi; grade 1, subepithelial space development, usually at the tip of a villus, with capillary congestion; grade 2, extension of subepithelial space with moderate lifting of the epithelial layer; grade 3, massive subepithelial lifting and increased vacuolar degeneration from the tips to the middle portions of villi; grade 4, denuded villi with lamina propria and dilated capillaries exposed; and grade 5, digestion and disintegration of the lamina propria, hemorrhage, and ulceration.

Two sections were prepared per animal, and ten randomly selected villi were graded in each section, giving 20 graded villi per animal. Grading was performed by two investigators who were blinded to group allocation, using a light microscope (BX43; Olympus, Tokyo, Japan) at a magnification of ×100, and the mean of the 20 gradings was used as that animal’s injury score. Individual scores for every animal are provided in S3 Data.

### Experiment 2

Mice (n = 19) were randomly assigned to SED (n = 5), EX1 (n = 5), EX2 (n = 4), or EX3 (n = 5). Exercise groups completed 1, 2, or 3 weeks of running, respectively, after 1 week of acclimation using the Experiment 1 protocol; SED mice did not undergo treadmill running. The day after the final session, mice were euthanized and jejunal tissues were collected. Jejunal HSP70, Bcl-2, LC3, and p62 expression was measured by western blotting because the exercise-associated cytokine pattern in Experiment 1 was more evident in the jejunum.

### Western blotting

Total protein was extracted by homogenizing jejunal tissue on ice in RIPA buffer (Thermo Fisher Scientific, cat. no. 89901) containing a protease inhibitor cocktail (Roche, cat. no. 11697498001). Protein concentrations were measured by the Lowry method. Protein (20 μg per lane) was separated by SDS-PAGE at room temperature and transferred to PVDF membranes at 4°C. Membranes were blocked in 5% skim milk for 20 min at room temperature and then incubated overnight at 4°C with primary antibodies against HSP70 (Enzo Life Sciences, cat. no. ADI-SPA-810-F, 1:1000), Bcl-2 (Santa Cruz Biotechnology, cat. no. sc-7382, 1:1000), LC3 (MBL, cat. no. M186-3, 1:500), p62 (Thermo Fisher Scientific, cat. no. PA5–20839, 1:1000) or β-actin (MBL, cat. no. PM053, 1:5000). After washing with TBST, membranes were incubated for 1 h at room temperature with goat anti-mouse IgG (H + L)-HRP conjugate (Bio-Rad, cat. no. 1706516, 1:5000) or goat anti-rabbit IgG (H + L)-HRP conjugate (Bio-Rad, cat. no. 1706515, 1:5000). After further washing with TBST, bands were detected with an enhanced chemiluminescence reagent (Bio-Rad, cat. no. 1705062) on a Molecular Imager ChemiDoc XRS+ (Bio-Rad).

Band volumes were quantified from the original, unadjusted 16-bit acquisition images using Image Lab software version 6.0, before any figure preparation. Each ratio was calculated as the band volume divided by the corresponding β-actin band volume, and the individual values are provided in S1 Data.

Before quantification, all membranes were examined for signal saturation. The HSP70, Bcl-2 and p62 bands were not saturated. The LC3 membranes were saturated in the LC3-II band in every archived exposure, and the LC3-II/I ratio was therefore not used as a quantitative outcome. The LC3 membrane images and the corresponding band volumes are provided in S1 File and S1 Data.

### Statistical analysis

Continuous data are presented as mean ± standard error of the mean and were assessed for normality using the Shapiro–Wilk test.

In Experiment 1, each cytokine was analyzed separately for the jejunum and the ileum using two-way analysis of variance, with exercise condition (sedentary versus exercise) and time after reperfusion (3 versus 6 h) as independent factors. Main effects and the exercise-by-time interaction were evaluated, and Sidak-adjusted post hoc comparisons assessed the exercise versus the sedentary group at each time point. Cytokine concentrations below the lower limit of detection were recorded as 0 pg/mL and retained as 0 in the analyses. Histological injury scores were analyzed with the same two-way model, and between-group comparisons at each time point were made using Student’s t test. Because the jejunal and ileal scores were obtained from the same animals, whether the degree of protection differed between the two segments was assessed as the group-by-segment interaction in a linear mixed model with animal as a random effect, fitted by maximum likelihood.

Experiment 2 comprised four graded durations of training (0, 1, 2, and 3 weeks), and two complementary analyses are reported. First, simple linear regression across all 19 animals assessed the association between training duration and the HSP70/β-actin, Bcl-2/β-actin, and p62/β-actin ratios; this analysis uses the whole cohort and corresponds to the graded design of the experiment. Second, each exercise group was compared with the sedentary group using Student’s t test with Bonferroni adjustment for three comparisons, and P < 0.017 (0.05/3) was considered significant; because each group comprised only four or five animals, these comparisons have limited statistical power and are reported alongside, rather than in place of, the regression analysis. No formal a priori power calculation was performed for Experiment 2; group sizes were based on the previous experimental framework and feasibility. Densitometric values below the limit of detection were recorded as 0 and retained as 0, and a sensitivity analysis excluding these values is provided in S1 Table.

P < 0.05, or the adjusted threshold where applicable, was considered statistically significant. Analyses were performed using JMP Pro 16 for Windows (SAS Institute Inc., Cary, NC, USA).

## Results

### Histological intestinal injury after gut ischemia–reperfusion

Mucosal injury was graded by the Chiu classification in the jejunum and ileum of 22 mice from Experiment 1 (3 h, n = 6 per group; 6 h, n = 5 per group; Fig 1; individual scores in S3 Data). In the sedentary group, the mean injury score was 2.60 ± 0.50 at 3 h and 2.48 ± 0.20 at 6 h in the jejunum and 2.84 ± 0.34 at 3 h and 2.47 ± 0.30 at 6 h in the ileum, corresponding to Chiu grades 2 (extension of the subepithelial space with moderate lifting of the epithelial layer) to 3 (massive subepithelial lifting and increased vacuolar degeneration).

**Fig 1:**
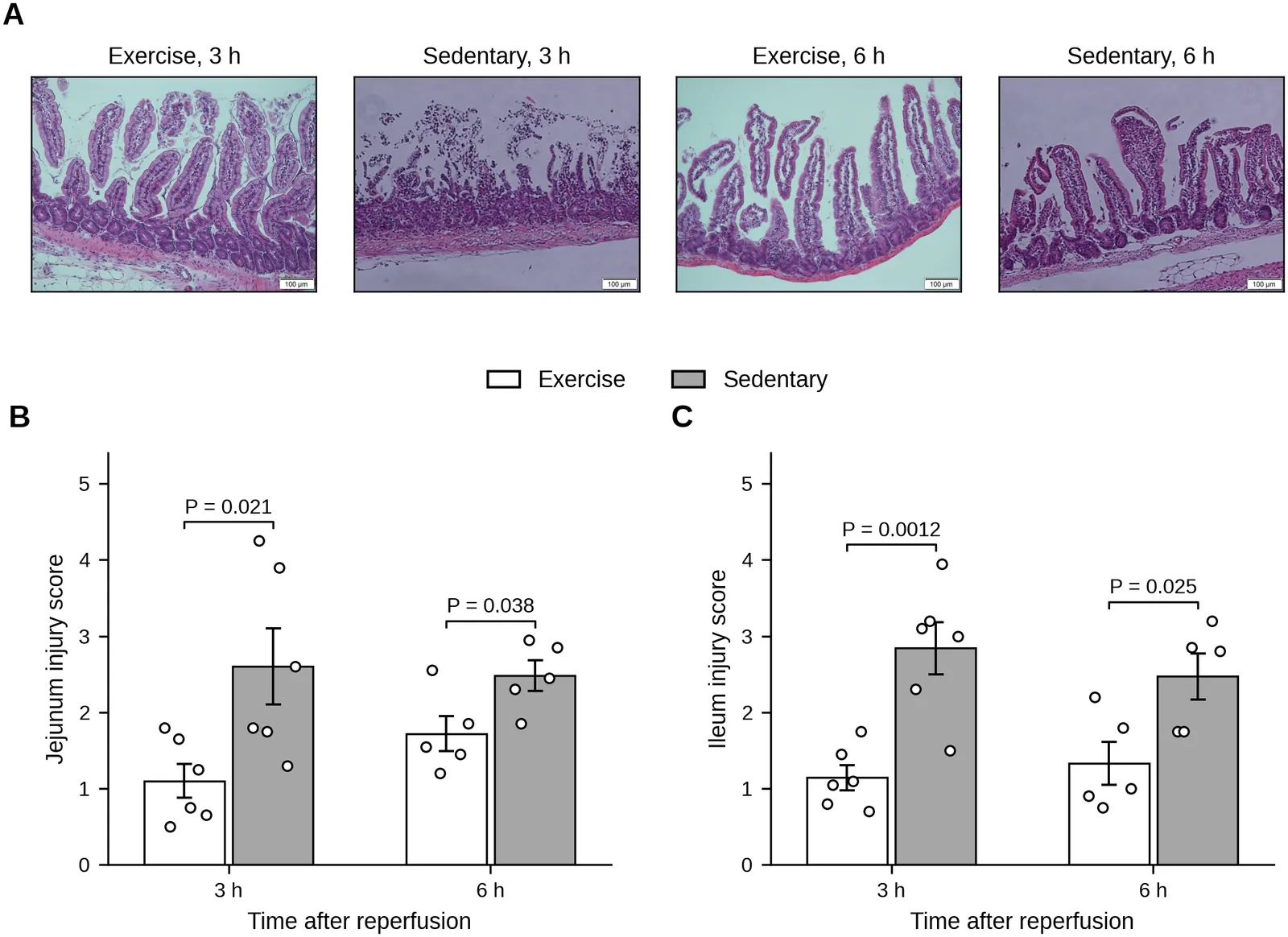
Histological intestinal injury after gut ischemia–reperfusion. (A) Representative jejunal sections (hematoxylin and eosin, × 100; scale bar 100 µm) from exercised and sedentary mice at 3 and 6 h after reperfusion (injury scores of the animals shown: exercise 3 h, 0.65; sedentary 3 h, 4.25; exercise 6 h, 1.85; sedentary 6 h, 2.95). Chiu injury scores in the (B) jejunum and (C) ileum. Bars are mean ± SE and circles are individual animals (3 h, n = 6 per group; 6 h, n = 5 per group). P values are for the between-group comparison at each time point; the main effect of exercise by two-way ANOVA was P = 0.0025 for the jejunum and P < 0.0001 for the ileum.

In the exercise group, the mean score was 1.10 ± 0.22 at 3 h and 1.72 ± 0.23 at 6 h in the jejunum and 1.14 ± 0.16 at 3 h and 1.33 ± 0.28 at 6 h in the ileum. Scores were lower in exercised mice at both time points in both segments (jejunum, P = 0.021 at 3 h and P = 0.038 at 6 h; ileum, P = 0.0012 at 3 h and P = 0.025 at 6 h). The main effect of exercise by two-way analysis of variance was P = 0.0025 for the jejunum and P < 0.0001 for the ileum.

Because the jejunal and ileal scores were obtained from the same animals, whether the degree of protection differed between the segments was assessed as the group-by-segment interaction in a linear mixed model with animal as a random effect. The interaction was not significant (P = 0.26); the reduction in injury did not differ between the two segments.

### Cytokine levels in the jejunum and ileum

Two-way analysis of variance of jejunal cytokine concentrations showed main effects of exercise on IL-12 (P = 0.023) and IL-6 (P = 0.024) and a main effect of time on MCP-1 (P = 0.0074). No exercise-by-time interaction was significant for any jejunal cytokine (all P > 0.05). Sidak-adjusted post hoc comparisons showed that IL-12 at 3 h (adjusted P = 0.042) and IL-6 at 6 h after reperfusion (adjusted P = 0.015) were lower in the exercise group than in the sedentary group.

In the ileum, main effects of exercise on IL-12 (P = 0.037) and of time on MCP-1 (P = 0.0028) and IL-6 (P = 0.036) were observed. No exercise-by-time interaction was significant for any ileal cytokine (all P > 0.05), and Sidak-adjusted post hoc comparisons showed no differences between the exercise and sedentary groups at either time point (Table 1).

**Table 1:** Jejunal and ileal cytokine concentrations after gut ischemia–reperfusion.

	3 h after reperfusion	6 h after reperfusion	P value				
	**Exercise**	**Sedentary**	**Exercise**	**Sedentary**	**Exercise**	**Time**	**Interaction**
***Jejunum***							
IL-12	0 ± 0*	5.94 ± 2.99	0 ± 0	2.35 ± 1.55	0.023	0.30	0.30
TNF-α	5.57 ± 2.31	5.87 ± 2.26	3.28 ± 1.46	5.83 ± 2.66	0.53	0.61	0.62
IFN-γ	5.23 ± 1.68	7.29 ± 1.63	2.80 ± 1.78	6.25 ± 2.02	0.14	0.34	0.70
MCP-1	49.2 ± 17.4	165 ± 59.0	312 ± 83.9	362 ± 114	0.30	0.0074	0.67
IL-10	5.03 ± 3.30	6.59 ± 3.00	2.10 ± 1.40	4.70 ± 2.21	0.43	0.36	0.84
IL-6	17.5 ± 6.55	24.3 ± 6.71	12.4 ± 3.55*	55.6 ± 17.8	0.024	0.21	0.091
***Ileum***							
IL-12	0 ± 0	2.96 ± 2.00	0 ± 0	3.05 ± 1.80	0.037	0.97	0.97
TNF-α	5.45 ± 2.34	7.08 ± 3.07	3.84 ± 1.65	5.88 ± 2.19	0.45	0.56	0.93
IFN-γ	3.39 ± 1.52	7.06 ± 1.54	2.82 ± 1.80	6.07 ± 1.99	0.058	0.66	0.90
MCP-1	84.8 ± 26.7	187 ± 47.7	352 ± 106	405 ± 78.2	0.29	0.0028	0.73
IL-10	5.83 ± 2.73	7.00 ± 2.29	9.17 ± 4.73	12.1 ± 4.54	0.59	0.27	0.82
IL-6	36.6 ± 12.5	54.3 ± 7.40	165 ± 58.1	151 ± 80.2	0.97	0.036	0.75

Values are pg/mL, mean ± SE; n = 6 per group at each time point. Concentrations below the lower limit of detection were recorded as 0 pg/mL and retained in the analyses. Each cytokine was analyzed separately for the jejunum and the ileum by two-way ANOVA with exercise condition and time after reperfusion as factors; the P values are for the two main effects and their interaction. *Sidak-adjusted P < 0.05 versus the sedentary group at the same time point (jejunal IL-12 at 3 h, adjusted P = 0.042; jejunal IL-6 at 6 h, adjusted P = 0.015). IFN-γ, interferon-γ; IL, interleukin; MCP-1, monocyte chemoattractant protein-1; TNF-α, tumor necrosis factor-α.

An exercise-associated reduction in IL-12 was thus present in both segments, whereas the reduction in IL-6 and the differences at individual time points were confined to the jejunum.

### HSP70, Bcl-2 and p62 expression in the jejunum

The jejunal HSP70/β-actin ratio was 0.072 ± 0.029 in SED, 0.270 ± 0.050 in EX1, 0.373 ± 0.051 in EX2 and 0.440 ± 0.092 in EX3 (Fig 2A), and training duration was positively associated with the ratio across all 19 animals (R² = 0.55, P = 0.0003; Fig 3A). All three exercise groups differed from SED in the group comparisons (P = 0.0094, 0.0010 and 0.0050 for EX1, EX2 and EX3).

**Fig 2:**
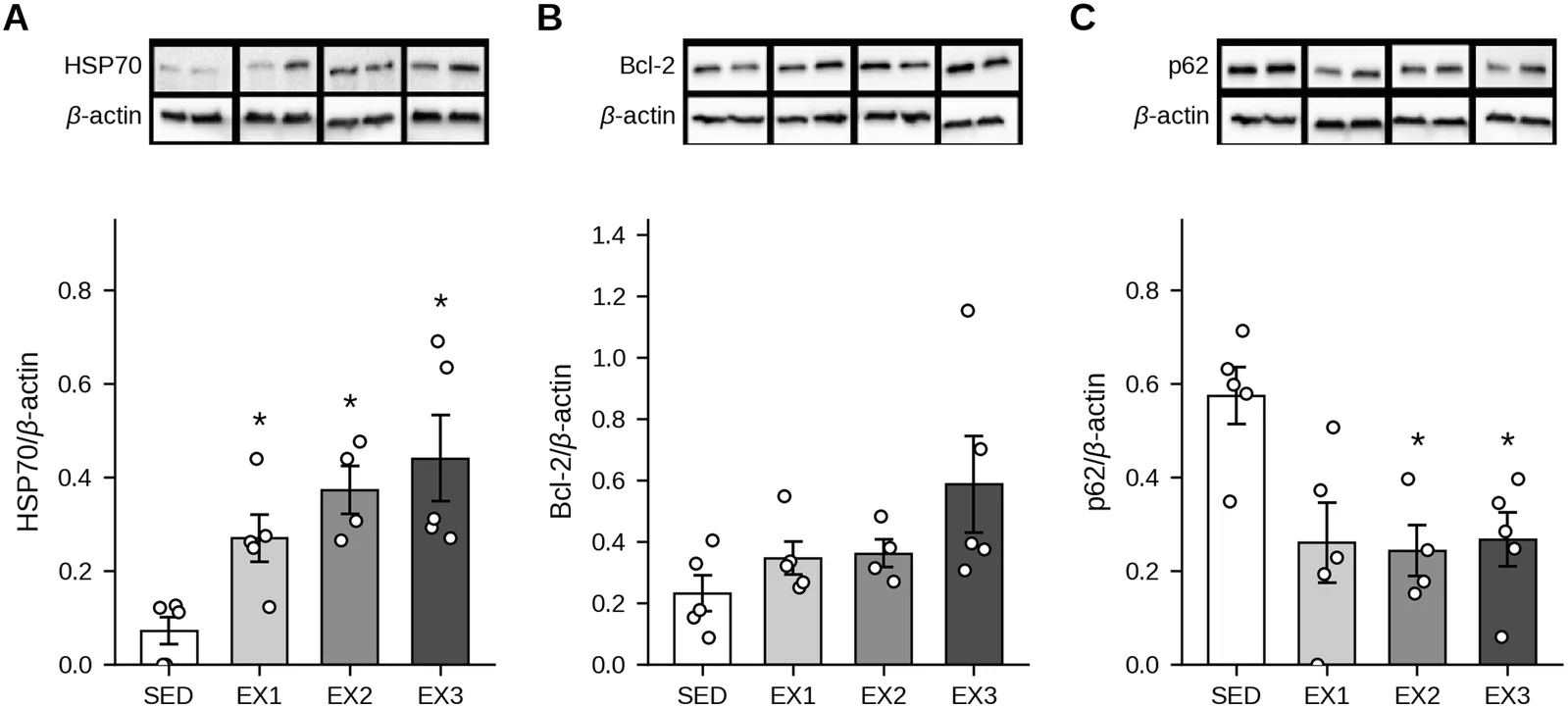
Jejunal HSP70, Bcl-2 and p62 expression before gut ischemia–reperfusion. Representative immunoblots and densitometry for (A) HSP70, (B) Bcl-2 and (C) p62 in sedentary mice (SED, n = 5) and after 1 (EX1, n = 5), 2 (EX2, n = 4) or 3 (EX3, n = 5) weeks of treadmill running. Bars are mean ± SE and circles are individual animals. *P < 0.017 versus SED (Student’s t test with Bonferroni correction for three comparisons). The lanes shown are representative; for HSP70 they were rearranged into group order from a single membrane, and for Bcl-2 and p62 they were taken from more than one membrane; the dividing lines mark these boundaries. Original images are provided in S1 File.

**Fig 3:**
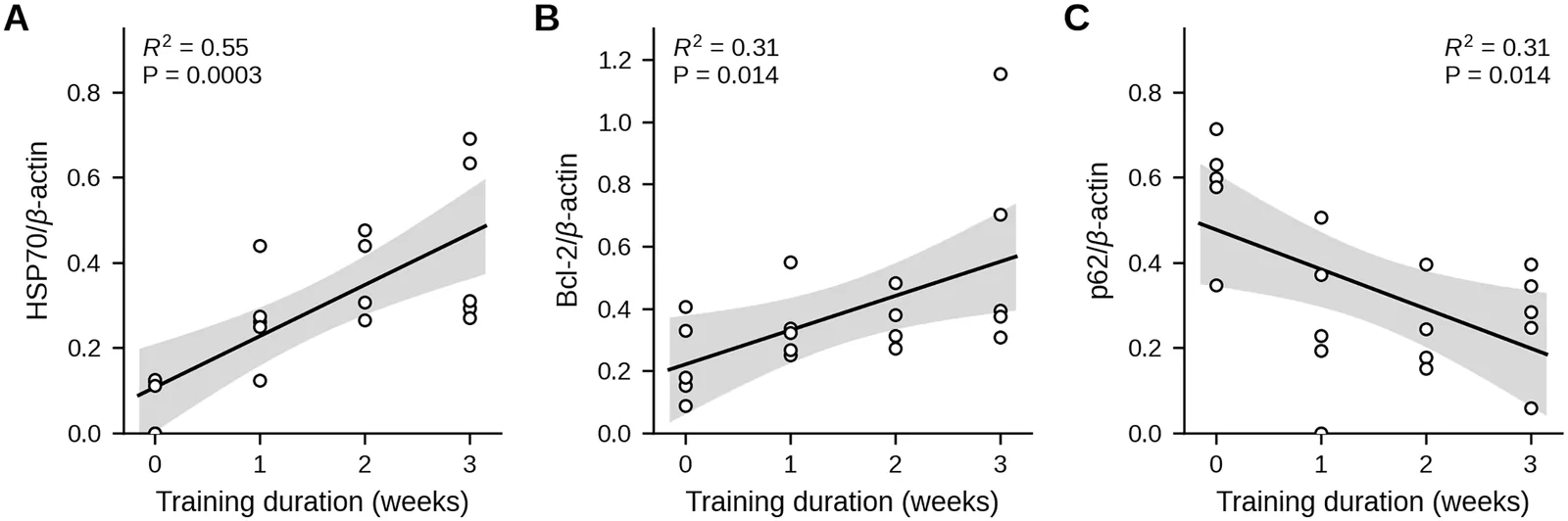
Treadmill-training duration and jejunal protein expression. Linear regression across all 19 animals for (A) HSP70/β-actin, (B) Bcl-2/β-actin and (C) p62/β-actin. Circles are individual animals; the line is the fitted regression with its 95% confidence interval.

The Bcl-2/β-actin ratio was 0.231 ± 0.059 in SED, 0.346 ± 0.053 in EX1, 0.362 ± 0.046 in EX2 and 0.587 ± 0.158 in EX3. Training duration was positively associated with the ratio (R² = 0.31, P = 0.014; Fig 3B), whereas none of the individual group comparisons reached the adjusted threshold (P = 0.19, 0.14 and 0.067; Fig 2B).

The p62/β-actin ratio was 0.574 ± 0.061 in SED and was lower in EX2 and EX3 (0.243 ± 0.055 and 0.267 ± 0.058; P = 0.0057 and 0.0065; Fig 2C); in EX1 it was 0.261 ± 0.086, a difference from SED that did not reach the adjusted threshold (P = 0.018). Training duration was negatively associated with the ratio (R² = 0.31, P = 0.014; Fig 3C), and the ratio in EX1 did not differ from that in EX3 (P = 0.95).

Because each group comprised only four or five animals, the group comparisons have limited power, and the regression analyses, which use all 19 animals, are reported alongside them. Values below the limit of detection were retained as 0; in the sensitivity analysis excluding these values (S1 Table), the regressions for HSP70 (P = 0.0033) and p62 (P = 0.0029) remained significant.

The LC3-II/I ratio was not used as a quantitative outcome because of membrane saturation (see Methods; S1 File and S1 Data).

## Discussion

We have previously reported that, in this model, treadmill running before gut ischemia–reperfusion (I/R) improves survival and attenuates organ injury and systemic inflammatory responses [6,7]. If prehabilitation acts on the intestine as ischemic preconditioning, the expression of cytoprotective proteins in the trained intestine should already be increased before injury, in proportion to the number of training sessions. Experiment 1 confirmed that gut I/R produced mucosal injury in the present cohorts and that this injury was attenuated by exercise; Experiment 2 tested the prediction, and the findings were consistent with it.

Mucosal injury was milder in exercised mice in both the jejunum and the ileum at both time points, and the degree of protection did not differ between the segments: the intestine itself was protected. The inflammatory response showed the same pattern. The exercise-associated reduction in IL-12 was present in both segments, whereas the reduction in IL-6 and the differences at individual time points were confined to the jejunum. MCP-1 rose between 3 and 6 h in both segments, indicating that the inflammatory response progressed with time after reperfusion in these cohorts. Because the exercise-associated changes were more evident in the jejunum, Experiment 2 examined that segment.

To test the prediction, we varied the training period over 1, 2 and 3 weeks (three, six and nine sessions) and examined intestine that had not undergone I/R. HSP70 was already higher than in sedentary mice after 1 week and increased further with training duration. p62 was lower after 2 and 3 weeks; after 1 week it was already at a similar level, although that comparison did not reach the adjusted threshold. Bcl-2 was associated with training duration but did not differ from the sedentary group in the individual group comparisons. The protein state of the intestine had therefore already changed after three sessions, and HSP70 was the protein that responded quantitatively to further training. HSP70 is a stress-response protein with cytoprotective and anti-apoptotic functions, and Bcl-2 contributes to the regulation of apoptosis [17,18]. p62 is degraded by autophagy [19]; LC3, however, could not be quantified in this study and autophagic flux was not assessed, so we draw no conclusion as to whether autophagy was increased.

Two accounts of how exercise protects the intestine are possible: exercise may attenuate the response to ischemia–reperfusion itself, or it may alter the intestine before the insult. Under the first, a difference would appear only after ischemia–reperfusion; under the second, a difference would be found in intestine that had not been injured. Both experiments were performed on the day after the final session, so Experiment 2 examined uninjured intestine at the same time point at which the intestine in Experiment 1 underwent ischemia. A difference was present: the trained intestine was already in a different state before the insult, which is consistent with preconditioning. Which part of that difference confers protection is not identified: no animal in this study provided both protein expression and an injury score, and we cannot distinguish whether the protein changes cause protection or accompany it.

This study confirmed, in the present cohorts, that prehabilitation protects the intestine itself from ischemia–reperfusion injury, and showed that this intestine differs in cytoprotective protein expression before injury; for HSP70, the difference followed the duration of training. Both findings are consistent with the hypothesis that prehabilitation acts on the intestine as ischemic preconditioning. Testing that hypothesis will require simultaneous measurement of protein expression and injury in the same animals, loss of protection when HSP70 induction is blocked, and direct assessment of autophagic flux.

Prehabilitation in clinical practice is undertaken to improve cardiopulmonary fitness and skeletal muscle mass, and its value has been understood as an increase in whole-body reserve [1–5]. The present findings indicate that the intestine itself may also be a target of exercise. The preoperative interval is usually a few weeks, within which a clear gain in fitness is difficult to achieve; here, the protein changes were already present after three sessions. If tolerance acquired at the level of the organ contributes to protection, short programs that do not appreciably improve fitness may still be of value. Because gut I/R promotes local inflammation and contributes to remote-organ injury [20–22], whether preoperative exercise increases intestinal tolerance in patients at risk of gut I/R [8] is worth testing. Future work should relate protein expression to injury within the same animals and determine, in larger animal models and in humans, whether preoperative exercise produces comparable intestinal changes and whether those changes can be detected clinically.

### Limitations

This study has several limitations. The sample sizes were small, and the EX2 group in Experiment 2 comprised four animals. Cytokines and proteins were assessed in separate animal cohorts, and Experiment 2 did not include ischemia–reperfusion. Intestinal blood flow and markers of oxidative stress were not measured; reliable direct measurement of intestinal blood flow in mice is difficult in our experience. Assessment was limited to 3 and 6 h after reperfusion, to a single training regimen and to male mice, so no conclusion can be drawn about longer-term recovery, the dose–response to exercise intensity, sex differences or extrapolation to humans.

## Conclusion

In conclusion, mucosal injury after gut ischemia–reperfusion was milder, and the early jejunal inflammatory response smaller, in prehabilitated mice, and the jejunum before ischemia differed in the expression of cytoprotective and autophagy-related proteins according to the duration of training. These findings are consistent with ischemic preconditioning of the intestine.

## Supporting information

S1 File Original uncropped and unadjusted Western blot images (S1_raw_images). Source images underlying the HSP70, Bcl-2, p62 and β-actin blots in Fig 2, and the LC3 membrane images, with the corresponding figure panel, sample loading order and approximate molecular weight.
(PDF)

S1 Data Individual-level Western blot densitometry data. Band volumes, the corresponding β-actin band volumes, the resulting ratios and the densitometry record used for each protein, for all 19 mice of Experiment 2. LC3-I and LC3-II band volumes and the LC3-II/I ratio are provided for completeness; the LC3-II/I ratio was not used as a quantitative outcome.
(XLSX)

S2 Data Individual-level cytokine measurements. Jejunal and ileal IL-12, TNF-α, IFN-γ, MCP-1, IL-10 and IL-6 concentrations for the 24 mice underlying Table 1.
(XLSX)

S3 Data Individual-level histological injury scores. Chiu grades for the 20 villi graded in each intestinal segment of each of the 22 mice, with the section means and the animal injury scores underlying Fig 1.
(XLSX)

S1 Table Sensitivity analysis excluding densitometric values below the limit of detection. Group comparisons and simple linear regression for HSP70/β-actin and p62/β-actin with the values recorded as 0 excluded.
(DOCX)

## References

[1] Gillis C, Buhler K, Bresee L, Carli F, Gramlich L, Culos-Reed N, et al. Effects of nutritional prehabilitation, with and without exercise, on outcomes of patients who undergo colorectal surgery: A systematic review and meta-analysis. Gastroenterology. 2018;155(2):391–410. 10.1053/j.gastro.2018.05.012 29750973

[2] Weimann A, Braga M, Carli F, Higashiguchi T, Hübner M, Klek S, et al. ESPEN guideline: Clinical nutrition in surgery. Clin Nutr. 2017;36(3):623–50. doi: 10.1016/j.clnu.2017.02.013 28385477

[3] Fukuda Y, Yamamoto K, Hirao M, Nishikawa K, Nagatsuma Y, Nakayama T, et al. Sarcopenia is associated with severe postoperative complications in elderly gastric cancer patients undergoing gastrectomy. Gastric Cancer. 2016;19(3):986–93. doi: 10.1007/s10120-015-0546-4 26407875

[4] Heger P, Probst P, Wiskemann J, Steindorf K, Diener MK, Mihaljevic AL. A systematic review and meta-analysis of physical exercise prehabilitation in major abdominal surgery (PROSPERO 2017 CRD42017080366). J Gastrointest Surg. 2020;24(6):1375–85. doi: 10.1007/s11605-019-04287-w 31228083

[5] Barberan-Garcia A, Ubré M, Roca J, Lacy AM, Burgos F, Risco R, et al. Personalised prehabilitation in high-risk patients undergoing elective major abdominal surgery: A randomized blinded controlled trial. Ann Surg. 2018;267(1):50–6. doi: 10.1097/SLA.0000000000002293 28489682

[6] Higashizono K, Fukatsu K, Watkins A, Watanabe T, Noguchi M, Murakoshi S, et al. Appropriate treadmill exercise improves survival after gut ischemia reperfusion in mice. Clin Nutr Exp. 2019;25:36–41.

[7] Takahashi K, Higashizono K, Fukatsu K, Murakoshi S, Takayama H, Noguchi M, et al. Prehabilitation ameliorates gut ischemia reperfusion injury in mice. J Surg Res. 2023;282:71–83. doi: 10.1016/j.jss.2022.08.033 36257166

[8] Schütz A, Eichinger W, Breuer M, Gansera B, Kemkes BM. Acute mesenteric ischemia after open heart surgery. Angiology. 1998;49(4):267–73. doi: 10.1177/000331979804900404 9555929

[9] Murry CE, Jennings RB, Reimer KA. Preconditioning with ischemia: A delay of lethal cell injury in ischemic myocardium. Circulation. 1986;74(5):1124–36. doi: 10.1161/01.cir.74.5.1124 3769170

[10] Xie Y, Xiao J, Fu C, Zhang Z, Ye Z, Zhang X. Ischemic preconditioning promotes autophagy and alleviates renal ischemia/reperfusion injury. Biomed Res Int. 2018;2018:8353987. doi: 10.1155/2018/8353987 29607326 PMC5828321

[11] Yuan Y, Pan S-S, Shen Y-J. Cardioprotection of exercise preconditioning involving heat shock protein 70 and concurrent autophagy: A potential chaperone-assisted selective macroautophagy effect. J Physiol Sci. 2018;68(1):55–67. doi: 10.1007/s12576-016-0507-7 27928720 PMC10717675

[12] Song Y-J, Zhong C-B, Wang X-B. Heat shock protein 70: A promising therapeutic target for myocardial ischemia-reperfusion injury. J Cell Physiol. 2019;234(2):1190–207. doi: 10.1002/jcp.27110 30132875

[13] Knight KA, Moug SJ, West MA. Systematic review: The impact of exercise on mesenteric blood flow and its implication for preoperative rehabilitation. Tech Coloproctol. 2017;21(3):185–201. doi: 10.1007/s10151-017-1589-9 28243813 PMC5360832

[14] Qamar MI, Read AE. Effects of exercise on mesenteric blood flow in man. Gut. 1987;28(5):583–7. doi: 10.1136/gut.28.5.583 3596339 PMC1432887

[15] Li L, Tan J, Miao Y, Lei P, Zhang Q. ROS and autophagy: Interactions and molecular regulatory mechanisms. Cell Mol Neurobiol. 2015;35(5):615–21. doi: 10.1007/s10571-015-0166-x 25722131 PMC11486209

[16] Chiu CJ, McArdle AH, Brown R, Scott HJ, Gurd FN. Intestinal mucosal lesion in low-flow states. I. A morphological, hemodynamic, and metabolic reappraisal. Arch Surg. 1970;101(4):478–83. doi: 10.1001/archsurg.1970.01340280030009 5457245

[17] Hu C, Yang J, Qi Z, Wu H, Wang B, Zou F, et al. Heat shock proteins: Biological functions, pathological roles, and therapeutic opportunities. MedComm (2020). 2022;3(3):e161. doi: 10.1002/mco2.161 35928554 PMC9345296

[18] Arya R, Mallik M, Lakhotia SC. Heat shock genes - integrating cell survival and death. J Biosci. 2007;32(3):595–610. doi: 10.1007/s12038-007-0059-3 17536179

[19] Mukhopadhyay S, Panda PK, Sinha N, Das DN, Bhutia SK. Autophagy and apoptosis: Where do they meet?. Apoptosis. 2014;19(4):555–66. doi: 10.1007/s10495-014-0967-2 24415198

[20] Moore FA. The role of the gastrointestinal tract in postinjury multiple organ failure. Am J Surg. 1999;178(6):449–53. doi: 10.1016/s0002-9610(99)00231-7 10670850

[21] Biffl WL, Moore EE, Moore FA, Carl VS, Kim FJ, Franciose RJ. Interleukin-6 potentiates neutrophil priming with platelet-activating factor. Arch Surg. 1994;129(11):1131–6. doi: 10.1001/archsurg.1994.01420350029002 7979944

[22] Matsukawa A, Hogaboam CM, Lukacs NW, Lincoln PM, Strieter RM, Kunkel SL. Endogenous monocyte chemoattractant protein-1 (MCP-1) protects mice in a model of acute septic peritonitis: Cross-talk between MCP-1 and leukotriene B4. J Immunol. 1999;163(11):6148–54. doi: 10.4049/jimmunol.163.11.6148 10570305

