# Supplementary material for: Treadmill prehabilitation attenuates jejunal inflammation and enhances cytoprotective protein expression in murine gut ischemia–reperfusion
Source: PLoS One. 2026 Nov 4;21(11):e0362208. doi: 10.1371/journal.pone.0362208 (PMC13734595; doi:10.1371/journal.pone.0362208)
Supplement: Supplementary file 1 [file pone.0362208.s001.pdf]

S1 File (S1_raw_images)

Original Uncropped and Unadjusted Western Blot Images Underlying Fig 2

**Manuscript title:**	Treadmill prehabilitation attenuates jejunal inflammation and enhances cytoprotective protein expression in murine gut ischemia–reperfusion
**Manuscript number:**	PONE-D-26-07233R2
**Corresponding author:**	Kazuya Higashizono

This file contains the original uncropped and unadjusted Western blot images underlying the HSP70, Bcl-2, p62 and β-actin blots presented in Fig 2, and the LC3 membrane images. Each image is reproduced in full at its original resolution. For each image, the corresponding figure panel, the source file name, the sample loading order and the approximate molecular weight of the target protein are given.

Western blot image acquisition

Protein bands were detected using an enhanced chemiluminescence reagent (Bio-Rad, cat. no. 1705062) and captured using a Molecular Imager ChemiDoc XRS+ system (Bio-Rad). Band densitometry was performed using Image Lab software, version 6.0. The original source images were saved in TIFF format.

Fig 2A — HSP70

Source file: HSP SED EX3 EX1 EX2.tif
Sample loading order: SED–EX3–EX1–EX2
Target protein: HSP70, approximately 70 kDa

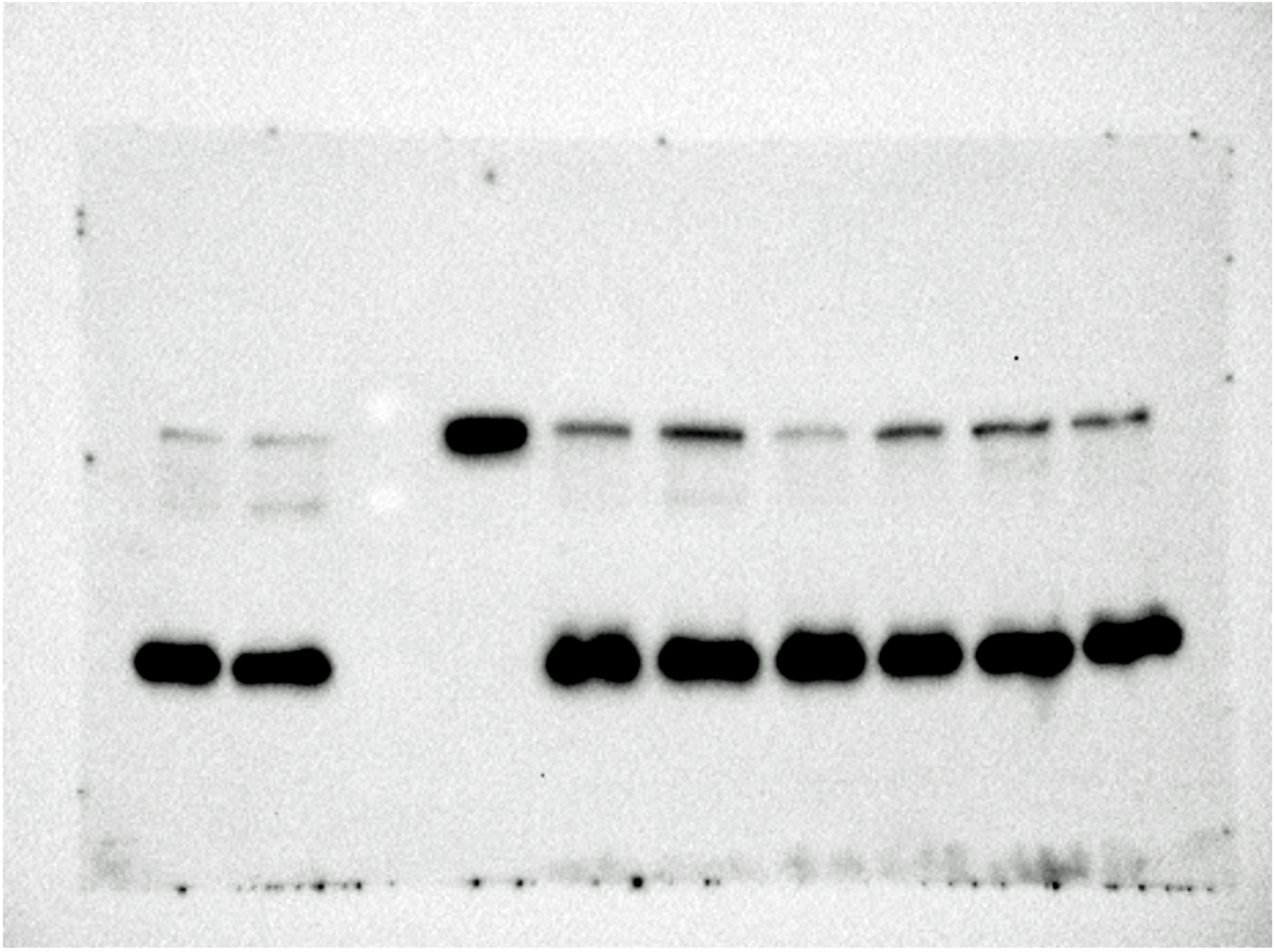

Figure assembly:
The samples were loaded in the order SED–EX3–EX1–EX2 so that strong signals in adjacent lanes would not affect each other. In Fig 2A, the lanes were rearranged into group order (SED–EX1–EX2–EX3); the rearranged boundaries are indicated by vertical dividing lines.

Fig 2A — β-actin (HSP70)

Source file: bactin SED EX3 EX1 EX2 for HSP70.tif
Sample loading order: SED–EX3–EX1–EX2
Target protein: β-actin, approximately 42 kDa

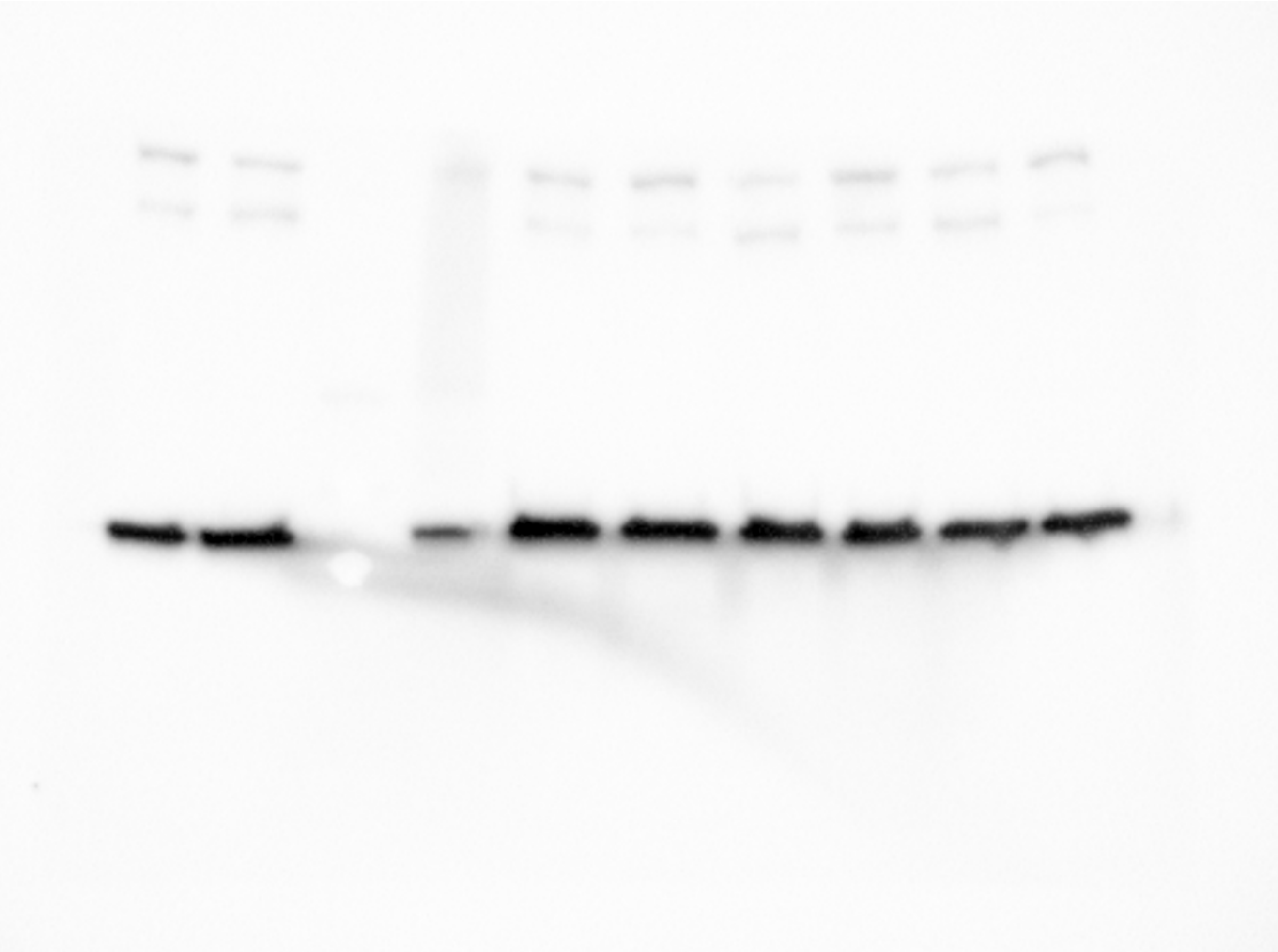

Figure assembly:
Lanes were rearranged in parallel with the corresponding HSP70 blot.

Fig 2B — Bcl-2: SED, EX1 and EX2

Source file: Bcl2 SED EX1 EX2.tif
Sample loading order: SED–EX1–EX2
Target protein: Bcl-2, approximately 26 kDa

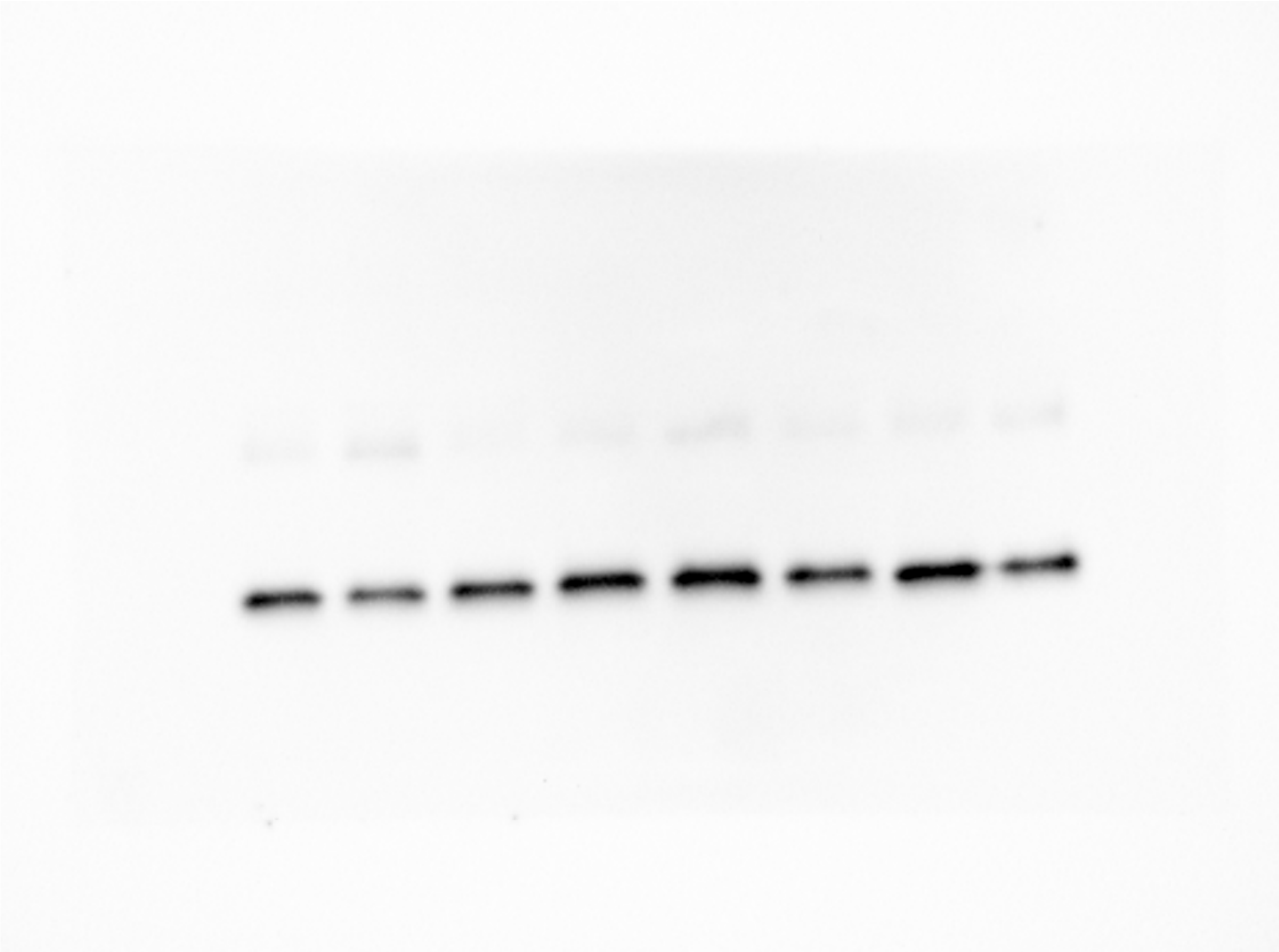

Fig 2B — Bcl-2: EX3

Source file: Bcl2 EX3.tif
Sample loading order: EX3
Target protein: Bcl-2, approximately 26 kDa

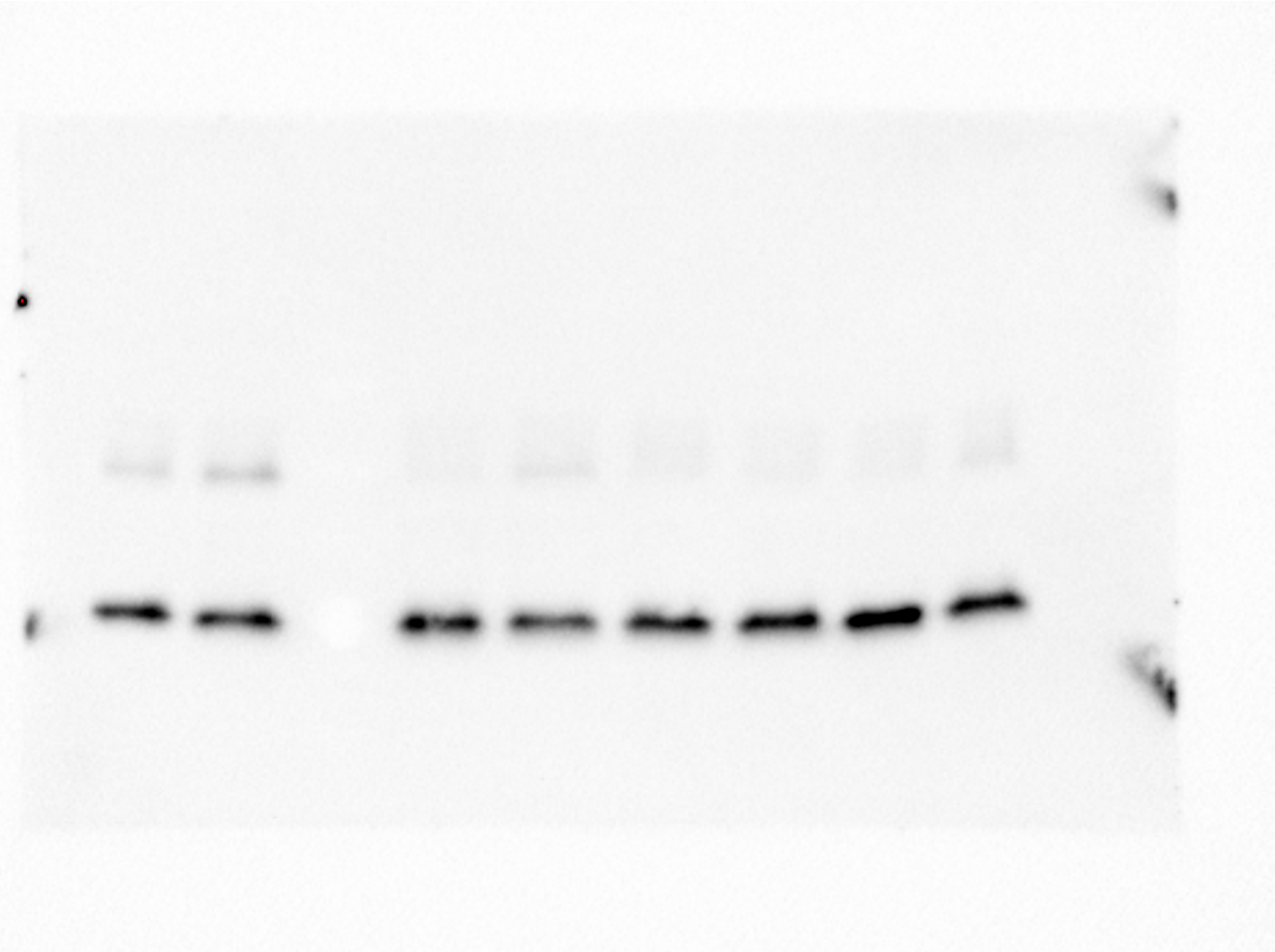

Fig 2C — p62: SED

Source file: p62 SED.tif
Sample loading order: SED
Target protein: p62, approximately 62 kDa

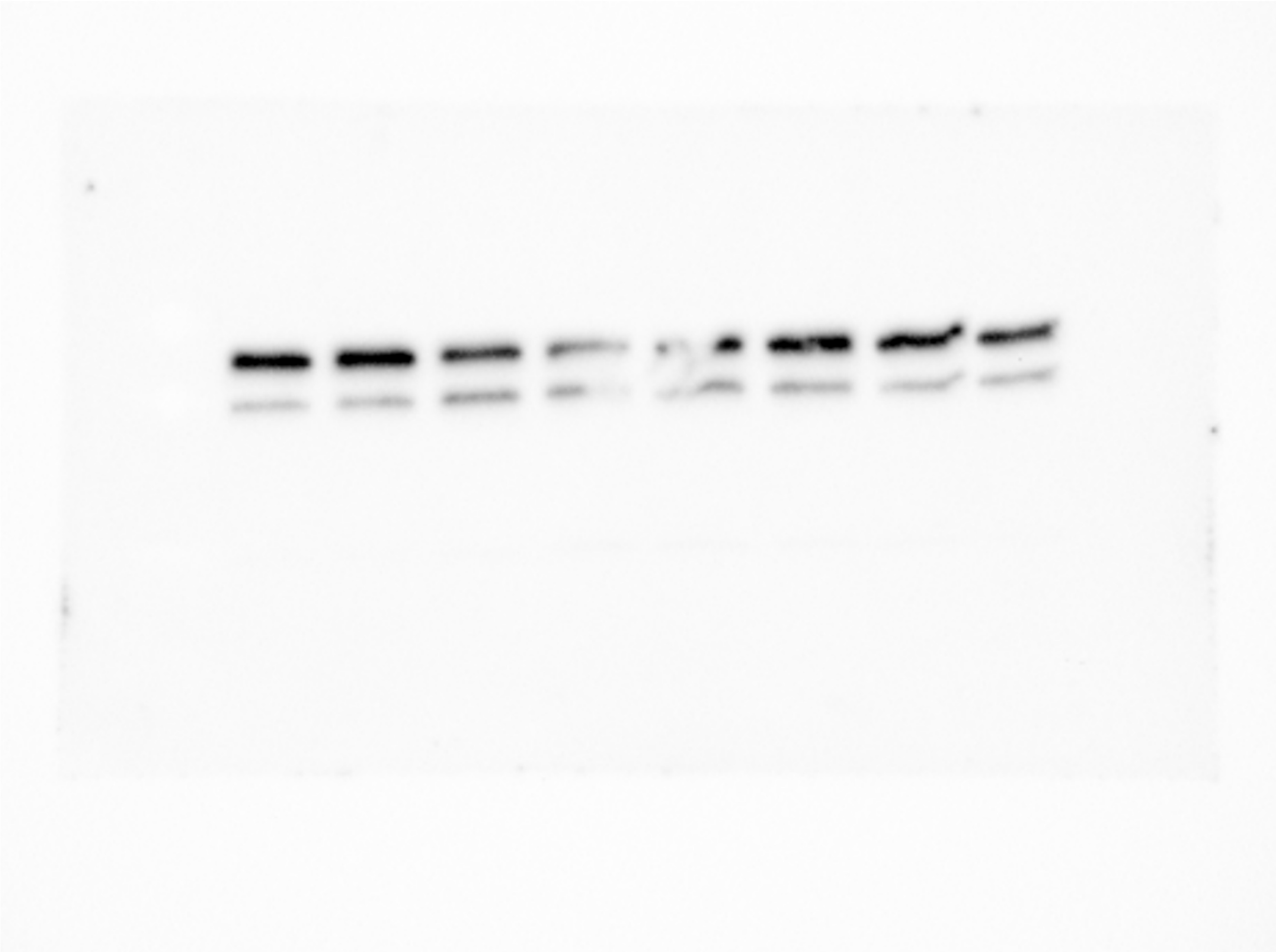

Fig 2C — p62: EX1 and EX2

Source file: p62 EX1 EX2.tif
Sample loading order: EX1–EX2
Target protein: p62, approximately 62 kDa

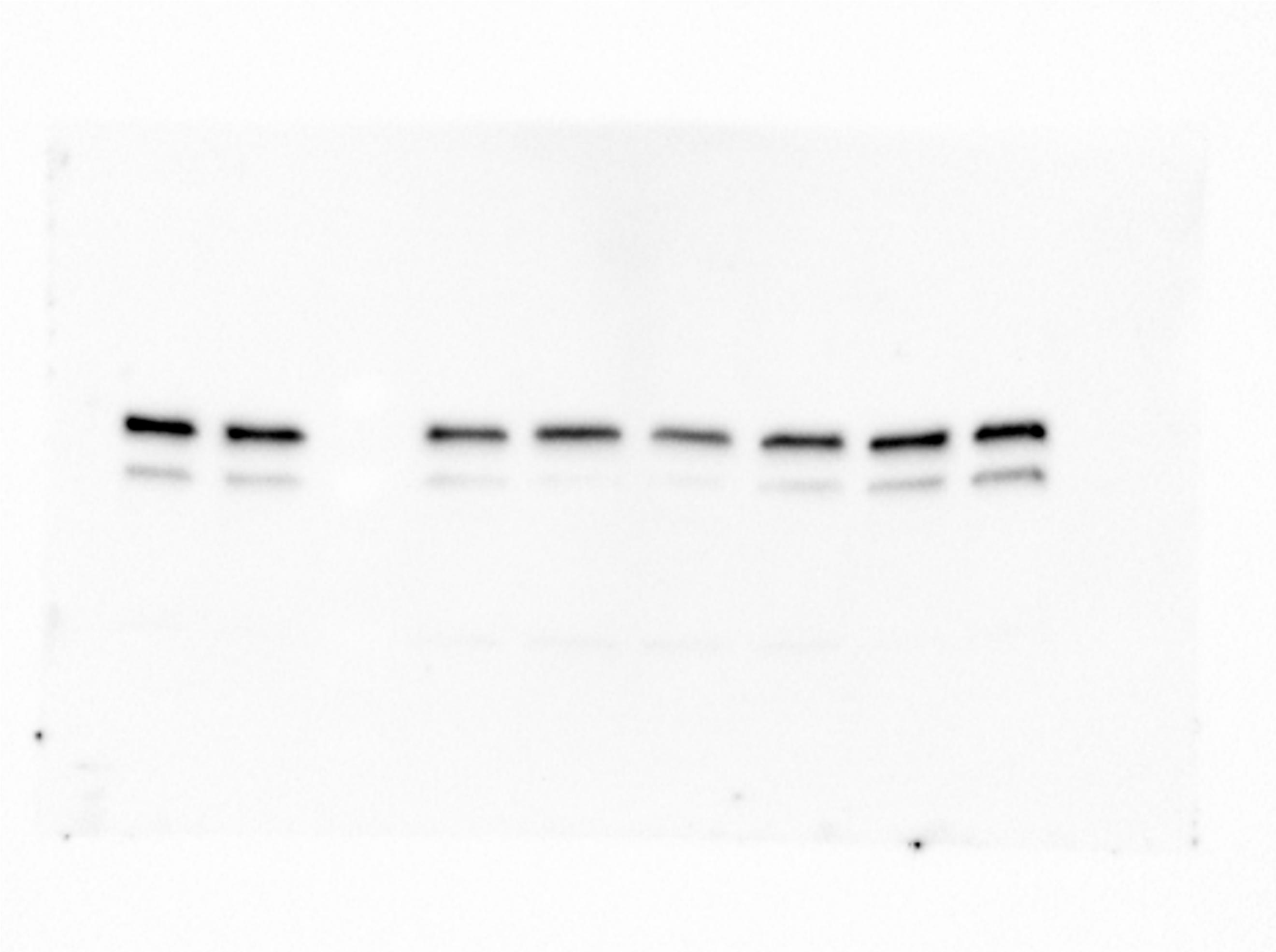

Fig 2C — p62: EX3

Source file: p62 EX3.tif
Sample loading order: EX3
Target protein: p62, approximately 62 kDa

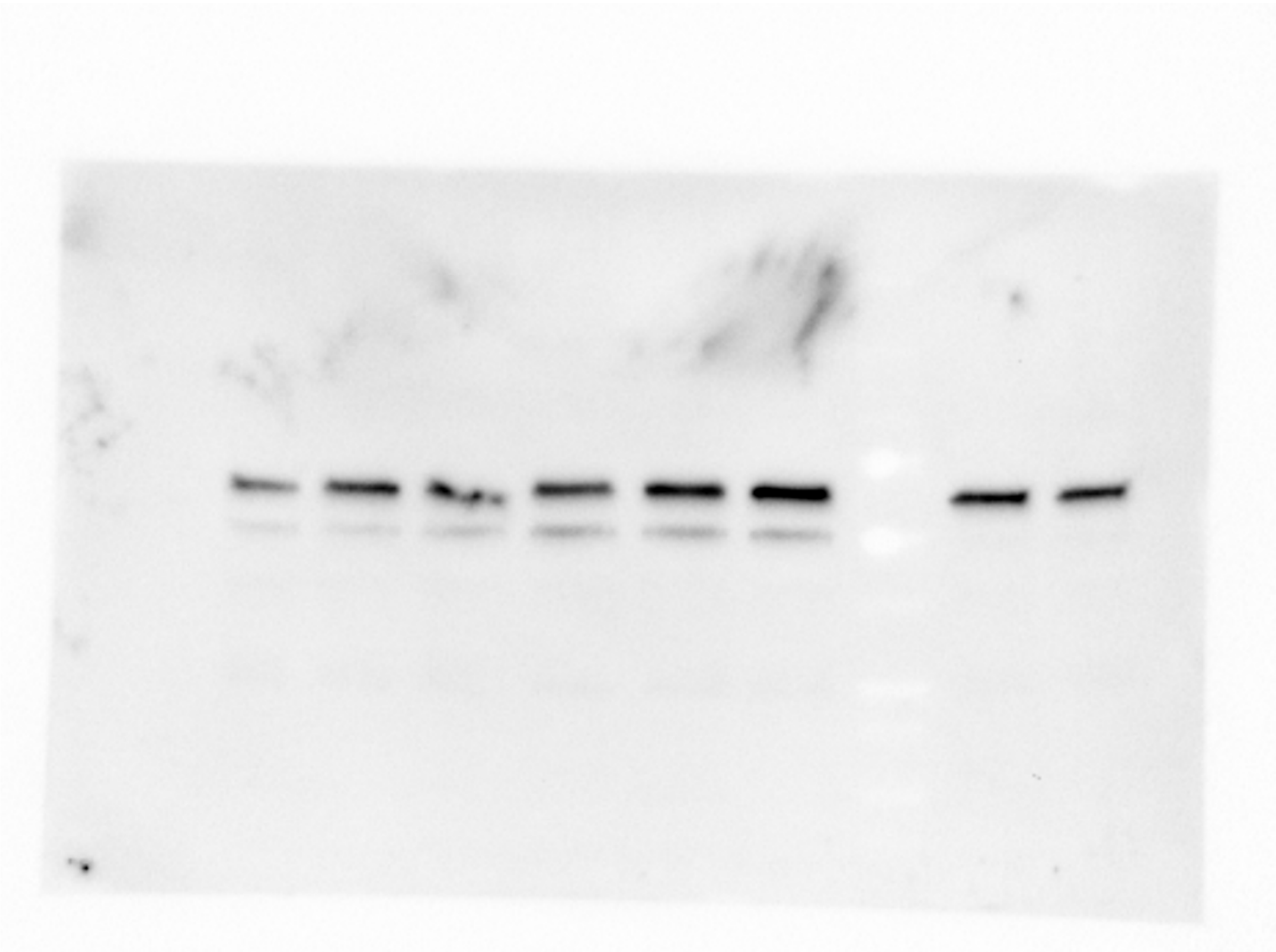

Fig 2B and 2C — β-actin (Bcl-2, p62)

Source file: bactin SED EX1 EX2 for Bcl2.tif
Target protein: β-actin, approximately 42 kDa

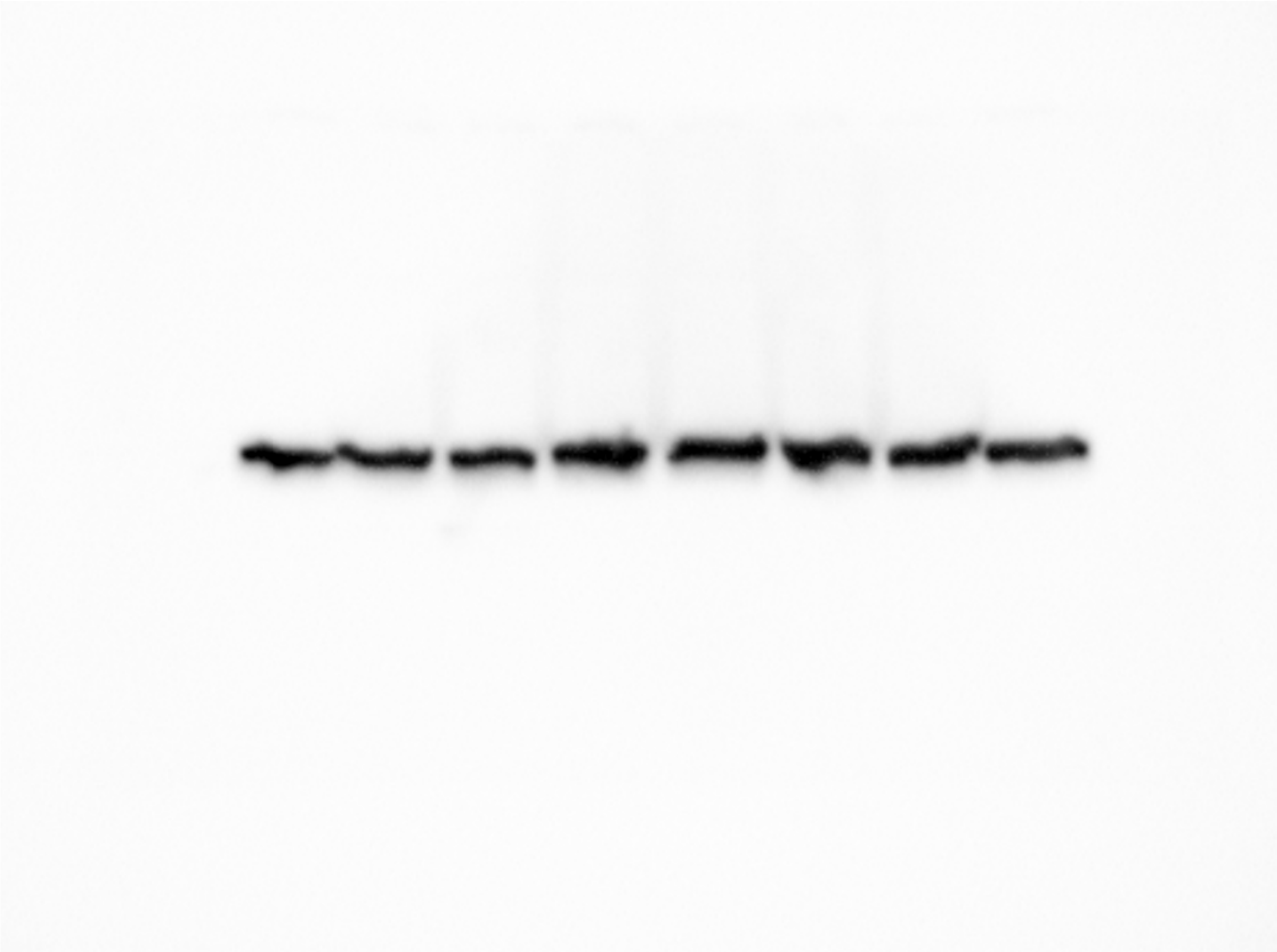

Fig 2B and 2C — β-actin (Bcl-2, p62)

Source file: bactin EX3 for Bcl2.tif
Target protein: β-actin, approximately 42 kDa

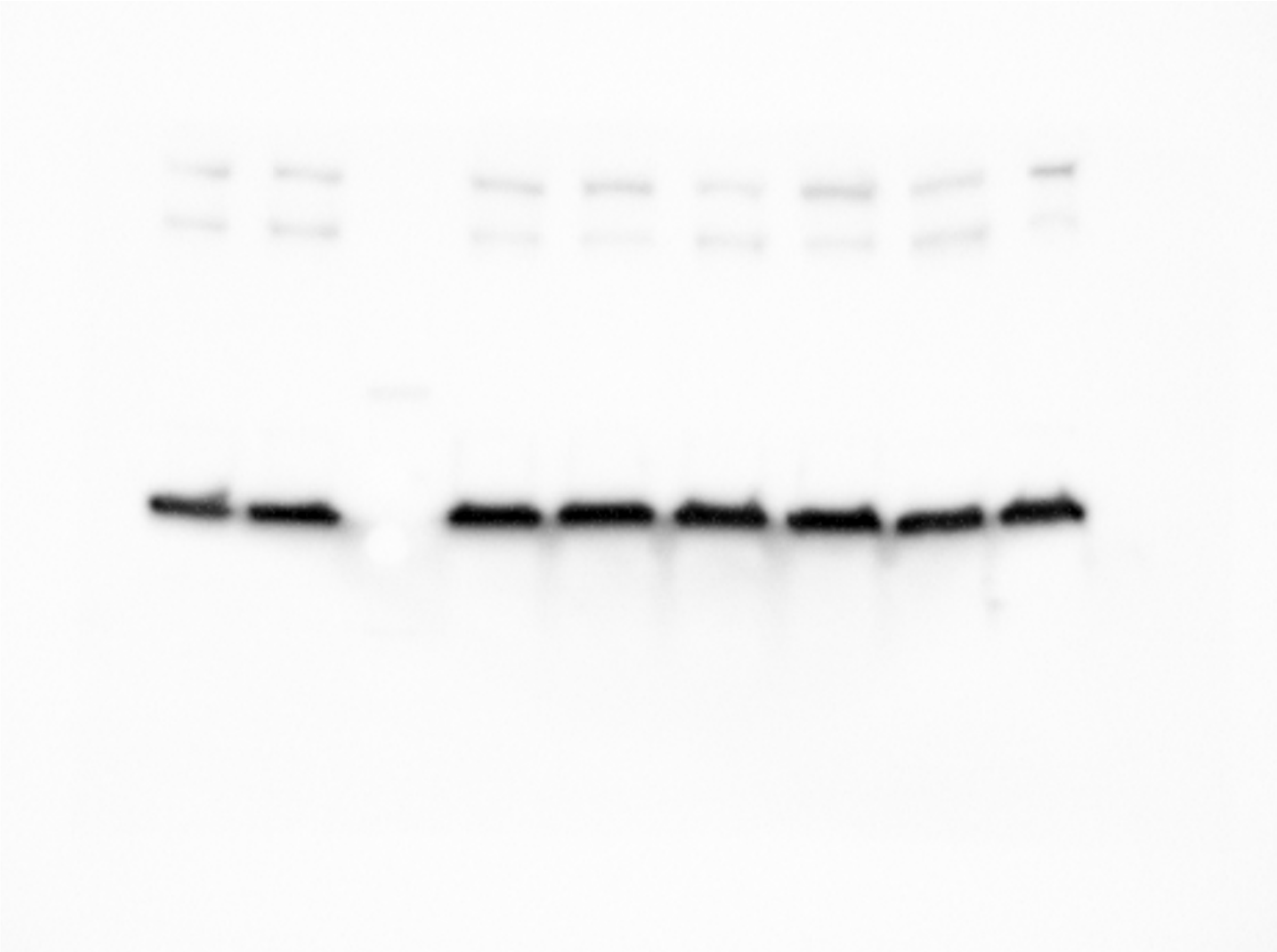

LC3: SED, EX1 and EX2 (not shown in the figures; S2 Data)

Source file: LC3 SED EX1 EX2.tif
Sample loading order: SED–EX1–EX2
Target protein: LC3-I, approximately 18 kDa; LC3-II, approximately 16 kDa

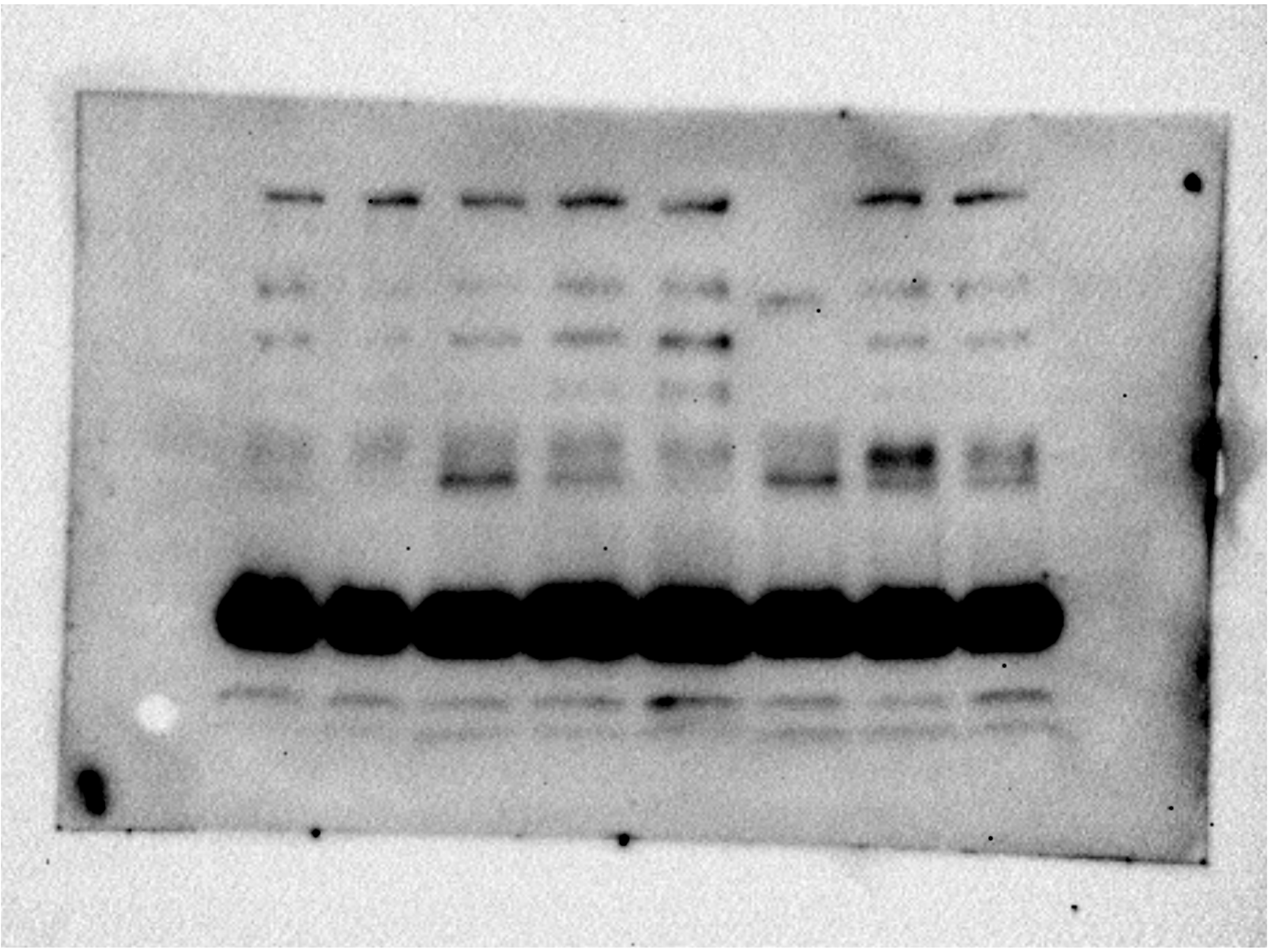

LC3: EX3 (not shown in the figures; S2 Data)

Source file: LC3 EX3.tif
Sample loading order: EX3
Target protein: LC3-I, approximately 18 kDa; LC3-II, approximately 16 kDa

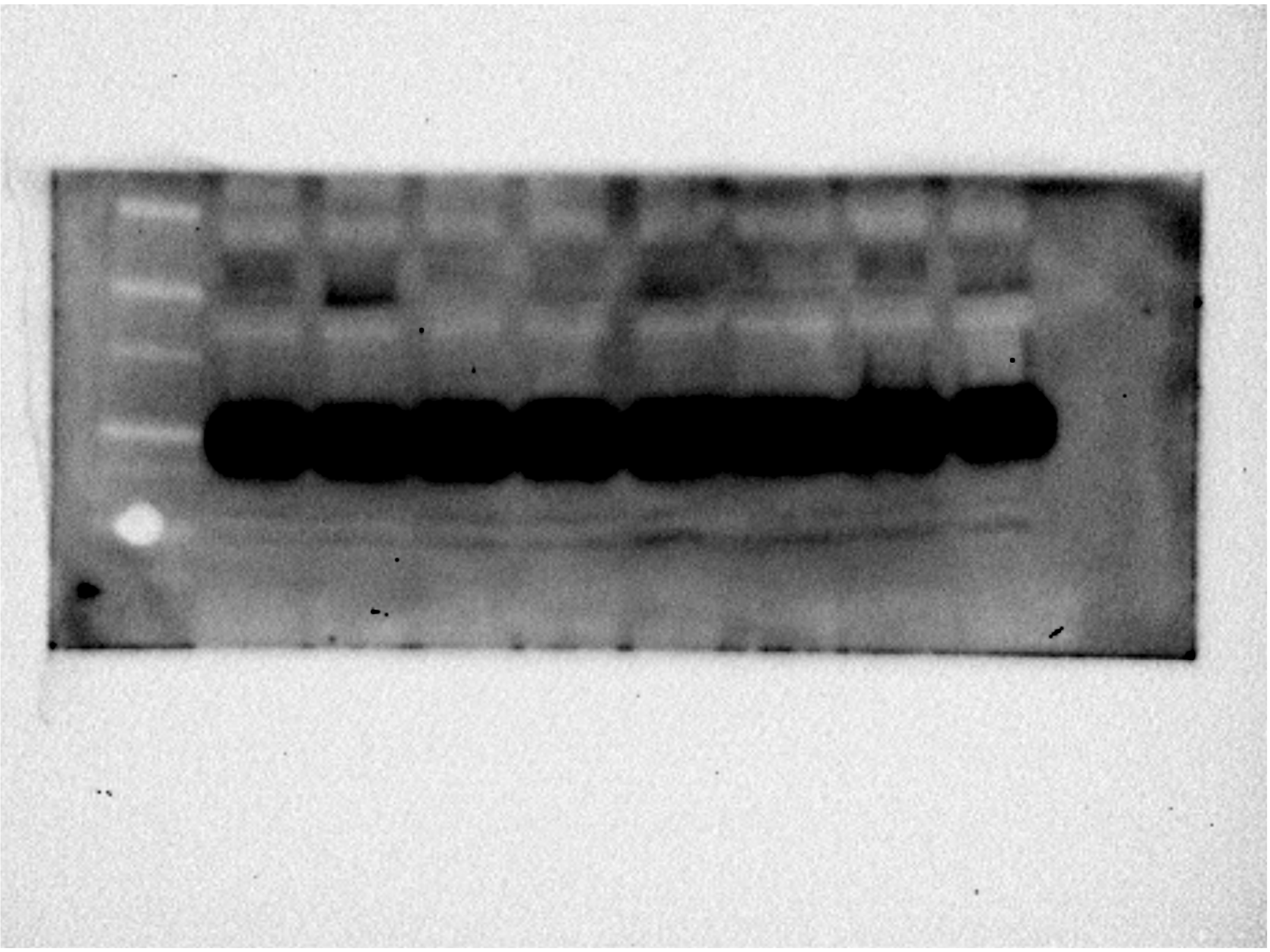

β-actin (LC3: SED, EX1 and EX2; not shown in the figures)

Source file: bactin SED EX1 EX2 for LC3.tif
Sample loading order: SED–EX1–EX2
Target protein: β-actin, approximately 42 kDa

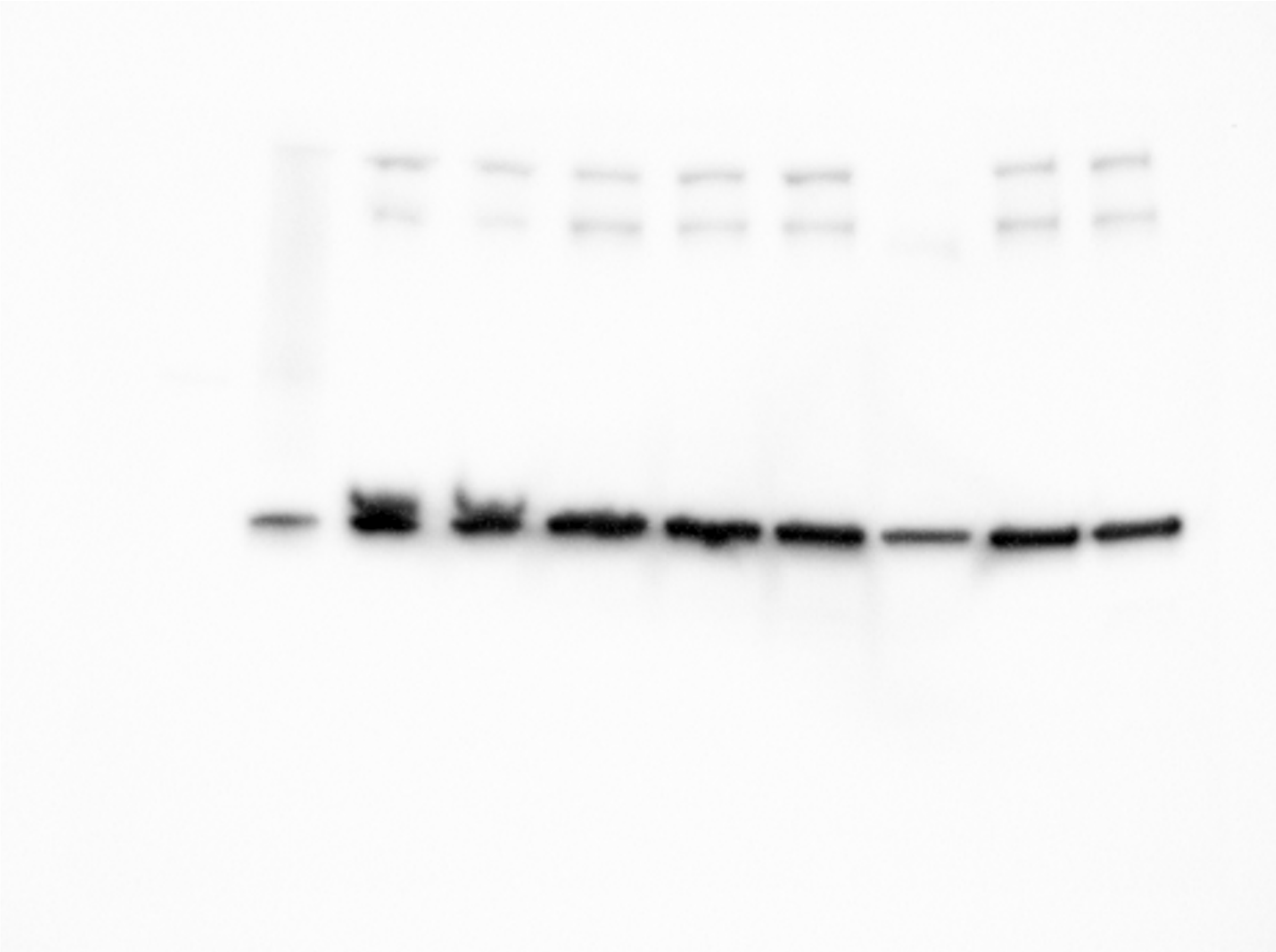

Correspondence between source images and Fig 2

Panel	Protein	Source file(s)	Loading order	Presentation in Fig 2
2A	HSP70	HSP SED EX3 EX1 EX2.tif	SED–EX3–EX1–EX2	Rearranged to SED–EX1–EX2–EX3
2A	β-actin	bactin SED EX3 EX1 EX2 for HSP70.tif	SED–EX3–EX1–EX2	Rearranged with HSP70
2B	Bcl-2	Bcl2 SED EX1 EX2.tif; Bcl2 EX3.tif	SED–EX1–EX2; EX3	Separated image sections
2C	p62	p62 SED.tif; p62 EX1 EX2.tif; p62 EX3.tif	SED; EX1–EX2; EX3	Separated image sections
2B, 2C	β-actin	bactin SED EX1 EX2 for Bcl2.tif; bactin EX3 for Bcl2.tif	—	Loading control for Bcl-2 and p62

Abbreviations: SED, sedentary mice; EX1, EX2 and EX3, mice examined after 1, 2 and 3 weeks of treadmill training; HSP70, heat shock protein 70; Bcl-2, B-cell lymphoma 2; LC3, microtubule-associated protein 1 light chain 3; p62, sequestosome 1.
