# Supplementary material for: Treadmill prehabilitation attenuates jejunal inflammation and enhances cytoprotective protein expression in murine gut ischemia–reperfusion
Source: PLoS One. 2026 Nov 4;21(11):e0362208. doi: 10.1371/journal.pone.0362208 (PMC13734595; doi:10.1371/journal.pone.0362208)
Supplement: Supplementary file 2 [file pone.0362208.s005.docx]

**S5 Table. Sensitivity analysis excluding densitometric values below the limit of detection.**

Densitometric values below the limit of detection were recorded as 0 and retained as 0 in the reported analyses (two animals for HSP70 and one animal for p62). This table repeats the group comparisons and the simple linear regression with those values excluded. Bcl-2 and LC3 contained no values below the limit of detection, so no sensitivity analysis applies to them. Individual values are provided in S2 Data.

| **Outcome** | **Analysis** | **n (SED/EX1/EX2/EX3)** | **EX1 vs SED** | **EX2 vs SED** | **EX3 vs SED** | **R²** | **P** |
| --- | --- | --- | --- | --- | --- | --- | --- |
| HSP70/β-actin | Reported analysis (0 retained) | 5/5/4/5 | 0.0094 | 0.0010 | 0.0050 | 0.55 | 0.0003 |
|  | Sensitivity analysis (0 excluded) | 3/5/4/5 | 0.067 | 0.0086 | 0.039 | 0.45 | 0.0033 |
| p62/β-actin | Reported analysis (0 retained) | 5/5/4/5 | 0.018 | 0.0057 | 0.0065 | 0.31 | 0.014 |
|  | Sensitivity analysis (0 excluded) | 5/4/4/5 | 0.033 | 0.0057 | 0.0065 | 0.44 | 0.0029 |

Group comparisons were made with Student’s t test with Bonferroni adjustment for three comparisons (P < 0.017, that is 0.05/3, considered significant). R² and P in the last two columns are for the simple linear regression of the ratio on training duration across all animals included in that analysis. With the values below the limit of detection excluded, the group comparisons for HSP70 in EX1 and EX3 no longer reach the adjusted threshold, whereas both regressions remain significant. The comparison for p62 in EX1 does not reach the adjusted threshold in either analysis.
